# Supplementary material for: Protein Kinase C (Pkc)-δ Mediates Arginine-Induced Glucagon Secretion in Pancreatic α-Cells
Source: Int J Mol Sci. 2022 Apr 4;23(7):4003. doi: 10.3390/ijms23074003 (PMC8999522; doi:10.3390/ijms23074003)
Supplement: Supplementary file 1 [file ijms-23-04003-s001.zip › Supplementary Figure/Supplementary Figure information.pdf]

Article

Protein kinase C (Pkc)-δ mediates arginine-induced glucagon secretion in pancreatic α-cells

Norikiyo Honzawa [1,3], Kei Fujimoto [2*], Masaki Kobayashi [3], Daisuke Kohno [3], Osamu Kikuchi [3], Hiromi Yokota-Hashimoto [3], Eri Wada [3], Yuichi Ikeuchi [3], Yoko Tabei [3], Gerald W. Dorn II [4], Kazunori Utsunomiya [1], Rimei Nishimura [1], and Tadahiro Kitamura [3*]

1 Division of Diabetes, Metabolism and Endocrinology, Department of Internal Medicine, Jikei University School of Medicine, 3-25-8 Nishishinbashi, Minato-ku, Tokyo 105-8461, Japan; mamnoon.am.m.m.h.m@gmail.com (N.H.); kazu-utsunomiya@jikei.ac.jp (K.U.); rimei@jikei.ac.jp (R.N.)

2 Division of Diabetes, Metabolism and Endocrinology, Department of Internal Medicine, Jikei University Daisan Hospital, 4-11-1, Izumihoncho, Komae-shi, Tokyo 201-8601, Japan; fuji@jikei.ac.jp (K.F.)

3 Metabolic Signal Research Center, Institute for Molecular and Cellular Regulation, Gunma University, 3-39-15 Showa-machi, Maebashi, Gunma 371-8512, Japan; masakikobayashi@gunma-u.ac.jp (M.K.); daisuke.kohno@gunma-u.ac.jp (D.K.); m10702010@gunma-u.ac.jp (O.K.); hiyokota@gunma-u.ac.jp (H.Y-H.); wada@riem.nagoya-u.ac.jp (E.W.); m1920045@gunma-u.ac.jp (Y.I.); tabei-y@gunma-u.ac.jp (Y.T.); kitamura@gunma-u.ac.jp (T.K.)

4 Center for Pharmacogenomics and Division of Cardiology, Department of Internal Medicine, Washington University School of Medicine, St. Louis, MO 63110, USA; gdorn@dom.wustl.edu (G.W.D.)

* Correspondence: kitamura@gunma-u.ac.jp (T.K.); Tel.: +81-27-220-8849, fuji@jikei.ac.jp (K.F.); Tel.: +81-3-3480-1151

Supplementary Figure 1. Glucagon staining with rat monoclonal anti-glucagon antibody in GFP-positive α-cells in *Gcg*$^{gfp/+}$ and *Gcg*$^{gfp/gfp}$ mice. Pancreatic sections of *Gcg*$^{gfp/+}$ and *Gcg*$^{gfp/gfp}$ mice were stained with rat monoclonal anti-glucagon antibody. Although glucagon signals perfectly colocalized with GFP in the *Gcg*$^{gfp/+}$ mice, glucagon was undetected in GFP-positive α-cells in *Gcg*$^{gfp/gfp}$ mice, indicating a high specificity of glucagon antibody used in this study. Glucagon is shown in red and DAPI in blue. Scale bar represents 50 μm. These studies were performed under blinded analysis.

Supplementary Figure 2. Characterization of pancreatic α-cell-specific Pkcδ knockout mice. **a–c** Body weights (a) and blood glucose levels during intraperitoneal glucose (1 g/kg) tolerance test (b) and intraperitoneal insulin (0.75 U/kg) tolerance test (c) were similar to αPkcδKO and control mice. n = 10, each group. * $p < 0.05$; t-test. Data are expressed as a box plot. αPkcδKO indicates *Gcg*$^{CreERT2/+}$; *Prkcd*$^{foxed/floxed}$ mice and Cont indicates *Gcg*$^{+/+}$; *Prkcd*$^{floxed/floxed}$ mice. **d** Pancreatic sections of αPkcδKO and control mice were subjected to Immunohistochemistry with rat monoclonal anti-glucagon and anti-insulin antibodies. glucagon is shown in red, insulin is shown in green, and DAPI is shown in blue. Scale bars represent 30 μm. These studies were performed under blinded analysis. **e–g** Pancreatic α-cell area (e), β-cell area (f), and the percentage of the α-cell area against the β-cell area (g) were similar to αPkcδKO and control mice. n = 4, each group. * $p < 0.05$; t-test. Data are expressed as means ± SEM. αPkcδKO indicates *Gcg*$^{CreERT2/+}$; *Prkcd*$^{foxed/floxed}$ mice and Cont indicates *Gcg*$^{+/+}$; *Prkcd*$^{floxed/floxed}$ mice.

Supplementary Figure 3. Plasma insulin levels in pancreatic α-cell-specific Pkcδ knockout mice. **a-c** No significant differences in fasting (a), random-fed (b), and arginine-induced (c, 15 min after 3 g/kg arginine intraperitoneal administration) plasma insulin levels between αPkcδKO and control mice. n = 10, each group. * $p < 0.05$; t-test. Data are expressed as a box plot. αPkcδKO indicates *Gcg*$^{CreERT2/+}$; *Prkcd*$^{foxed/floxed}$ mice and Cont indicates *Gcg*$^{+/+}$; *Prkcd*$^{floxed/floxed}$ mice.
